# Reduced efficacy of selection in regions of the *Drosophila *genome that lack crossing over

**DOI:** 10.1186/gb-2007-8-2-r18

**Published:** 2007-02-06

**Authors:** Penelope R Haddrill, Daniel L Halligan, Dimitris Tomaras, Brian Charlesworth

**Affiliations:** 1 Institute of Evolutionary Biology, School of Biological Sciences, University of Edinburgh, Edinburgh, EH9 3JT, UK; 2 15 Smirnis St, 15669, Papagou, Athens, Greece

## Abstract

**Background:**

The recombinational environment is predicted to influence patterns of protein sequence evolution through the effects of Hill-Robertson interference among linked sites subject to selection. In freely recombining regions of the genome, selection should more effectively incorporate new beneficial mutations, and eliminate deleterious ones, than in regions with low rates of genetic recombination.

**Results:**

We examined the effects of recombinational environment on patterns of evolution using a genome-wide comparison of *Drosophila melanogaster *and *D. yakuba*. In regions of the genome with no crossing over, we find elevated divergence at nonsynonymous sites and in long introns, a virtual absence of codon usage bias, and an increase in gene length. However, we find little evidence for differences in patterns of evolution between regions with high, intermediate, and low crossover frequencies. In addition, genes on the fourth chromosome exhibit more extreme deviations from regions with crossing over than do other, no crossover genes outside the fourth chromosome.

**Conclusion:**

All of the patterns observed are consistent with a severe reduction in the efficacy of selection in the absence of crossing over, resulting in the accumulation of deleterious mutations in these regions. Our results also suggest that even a very low frequency of crossing over may be enough to maintain the efficacy of selection.

## Abstract 2

Observations from a genome-wide comparison of *Drosophila melanogaster *and *Drosophila yakuba *are consistent with a severe reduction in the efficacy of selection in the absence of crossing over, resulting in the accumulation of deleterious mutations in these regions.

## Background

Patterns of molecular evolution can be profoundly different between loci that differ in their recombinational environment. This is due to Hill-Robertson interference [1], whereby any locus linked to another that is under directional selection experiences a reduction in effective population size (N_e_). Because the efficacy of selection on a mutation is a function of the product of N_e _and the selection coefficient on a mutation (s), this linkage affects the probability of fixation of a new mutation [2]; favourable mutations are less likely to reach fixation, whereas the opposite is true for deleterious mutations. In other words, selection at one locus has the effect of increasing the effects of genetic drift at another, linked locus. Recombination reduces the effect of this interference, increasing N_e _and hence the efficacy of selection. We would therefore expect higher levels of adaptation, and lower rates of fixation of deleterious mutations, in genomic regions with high levels of genetic recombination, as compared with regions with little or no recombination [3,4].

Various studies have found evidence for such effects, primarily in nonrecombining genomes or chromosomes. For example, endosymbiotic bacteria experience small population sizes and minimal rates of recombination, resulting in accumulation of mildly deleterious mutations and possibly also reduced rates of adaptation [5-7]. The neo-sex chromosomes of *Drosophila miranda *have also provided compelling evidence for the effects of Hill-Robertson interference, showing elevated rates of fixation of deleterious mutations, and a reduced rate of adaptive evolution on the nonrecombining neo-Y chromosome, compared with the neo-X chromosome [8-11]. In addition to studies examining rates of evolution in nonrecombining chromosomes and genomes, investigation of different recombinational environments within the same genome or chromosome have proved fruitful. For example, in two studies of different recombination regions in *Drosophila*, Betancourt and Presgraves [12] and Presgraves [13] concluded that recombination affects the efficiency of selection on amino acid sequences, with reduced rates of adaptive evolution in regions of low recombination; these also experience higher frequencies of mildly deleterious segregating mutations [13].

However, these two studies used samples that represent only a small fraction of genes found in the *Drosophila *genome, which may also be biased toward genes that are known to be rapidly evolving, so that the results may not be entirely representative of the genome as a whole. In addition, it is currently unclear what proportion of amino acid differences between species is the result of positive selection. Some studies have found evidence that much protein evolution in *Drosophila *is the result of positive selection [14-16], but the generality of this result is still uncertain. The relative proportions of mutations that are advantageous as opposed to deleterious will be important in determining the influence of recombinational environment on patterns of evolution. The genomes of a number of *Drosophila *species have recently been sequenced, or are in the process of being sequenced, so that genome-wide comparisons are now possible. We use a dataset of more than 7,500 genes from *D. melanogaster *and the closely related species *D. yakuba *to examine the effects of recombinational environment on rates and patterns of evolution in coding and noncoding sequences, and on measures of adaptation at the molecular level.

## Results

The final dataset consisted of 7,612 genes, divided into recombination regions as follows: high crossover frequency (*n *= 3,859), intermediate crossover frequency (*n *= 2,555), low crossover frequency (*n *= 1,111), and no crossing over (*n *= 87). We also divided the no crossover category into fourth chromosome (*n *= 67) and non-fourth chromosome genes (*n *= 20), in order to examine whether there are any differences between no crossover genes on chromosomes with crossing over, and genes on a chromosome that is entirely crossover free. Sample sizes for the intron analyses were as follows: 10,407 in genes with high crossover frequency (6,474 short [≤80 base pairs (bp)] and 3,933 long [>80 bp]); 6,965 in genes with intermediate crossover frequency (4,445 short and 2,520 long); 2,898 in genes with low crossover frequency (1,800 short and 1,098 long); 218 in genes with no crossover (120 short and 97 long); 181 in fourth chromosome genes (96 short and 85 long); and 37 in non-fourth chromosome, no crossover genes (24 short and 13 long). We refer to crossing over rather than recombination, because there is evidence that gene conversion occurs in regions of the *D. melanogaster *genome with very low or zero frequencies of crossing over [17,18].

**Figure 1:**
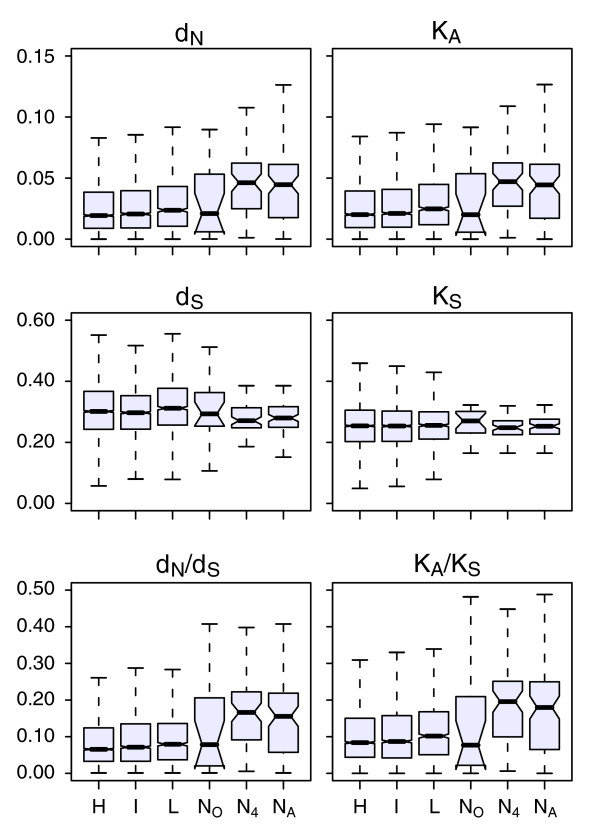
Notched box-plots of d_N_, d_S_, d_N_/d_S_, K_A_, K_S _and K_A_/K_S _for each recombination region. Shown are notched box-plots of d_N_, d_S_, d_N_/d_S_, K_A_, K_S _and K_A_/K_S _for regions of high (H), intermediate (I), and low (L) frequency of crossing over and regions of no crossing over, divided into non-fourth chromosome genes (N_O_), fourth chromosome genes (N_4_), and all no crossing over region genes (N_A_). The box extends from the lower to the upper quartile, with a line in the middle at the median. The dotted bars represent the 5th and 95th percentiles. The notches represent an estimate of the uncertainty about the medians for box-to-box comparison; when the notches for two samples do not overlap, the medians of the two groups differ at the 5% significance level.

We found a highly significant effect of recombinational environment on levels of the codon-based PAML measures of sequence divergence (see Materials and methods, below) d_N_, d_S_, and d_N_/d_S _(Kruskal-Wallis test: d_N_, H = 36.84, degrees of freedom [df] = 3, *P *< 10^-4^; d_S_, H = 40.03, df = 3, *P *< 10^-4^; and d_N_/d_S_, H = 38.16, df = 3, *P *< 10^-4^; Figure 1). The no crossover region exhibits elevated levels of d_N _and d_N_/d_S_, with median values being approximately double those found in other recombination regions, and, surprisingly, a somewhat reduced value for d_S_. To further investigate this, we used Gestimator (see Materials and methods, below) to calculate values of the nucleotide site based measures of divergence K_A _and K_S_. Although these results exhibit qualitatively the same patterns as the d_N _and d_S _values, recombinational environment exhibited a significant effect only on K_A _values and not on K_S _values (K_A_, H = 38.21, df = 3, *P *< 10^-4^; K_S_, H = 1.15, df = 3, *P *= 0.76; Figure 1).

Although pairwise tests indicate that there are some significant differences in divergence measures between high, intermediate, and low crossover regions (data not shown), the magnitude of these differences is extremely small compared with the difference between the no crossover region and the rest of the genome (Figure 1). The no crossover region is therefore the only region to show clear evidence of a distinctly different rate of nonsynonymous evolution, and there is an indication that it may also have a reduced rate of synonymous evolution.

However, when we examined differences between the two groups of genes within no crossover regions, namely the fourth chromosome and non-fourth chromosome genes, we found some surprising differences. Compared with the fourth chromosome genes, the non-fourth chromosome genes exhibit levels of nonsynonymous and synonymous evolution that are closer to those of the high, intermediate, and low crossover regions (Figure 1), and they are not significantly different from these regions (Wilcoxon rank sum test on d_N_, d_S_, d_N_/d_S_, K_A_, and K_S_; non-fourth versus high, intermediate and low combined: *P *> 0.34 in all cases) or the fourth chromosome genes (non-fourth versus fourth: *P *> 0.05 in all cases).

We also examined three measures of codon usage bias: effective number of codons (ENC), the frequency of optimal codons (Fop), and the GC content of the third position of codons (GC3; see Materials and methods, below, and Table 1). As expected from previous work [19,20], the no crossover region shows almost no evidence of codon usage bias, with elevated ENC and reduced Fop compared with the other recombination regions. Interestingly, the non-fourth chromosome genes within the no crossover category appear to exhibit levels of codon usage bias intermediate between the crossing over regions and the fourth chromosome genes. Betancourt and Presgraves [12] found that Fop was strongly negatively correlated with d_N _in their dataset but weakly positively correlated with d_S_. We find a significantly negative correlation between Fop and d_N _in all recombination regions (Spearman rank correlation [R_s_] with 95% confidence interval [CI; obtained by bootstrapping across genes]: high crossover R_s _= -0.436, 95% CI = -0.463 to -0.409; intermediate crossover R_s _= -0.476, 95% CI = -0.508 to -0.444; low crossover R_s _= -0.438, 95% CI = -0.487 to -0.383; no crossover R_s _= -0.228, 95% CI = -0.435 to -0.042), although the relationship is much weaker in the no crossover region. When the no crossover region is divided into fourth and non-fourth chromosome genes, the correlations are both still negative although not significantly so (fourth chromosome R_s _= -0.078, 95% CI = -0.346 to 0.166; non-fourth chromosome R_s _= -0.135, 95% CI = -0.616 to 0.350). However, the relationship with d_S _is less clear; the correlations are not significantly different from zero in high, intermediate, and no crossover regions (high R_s _= -0.001, 95% CI = -0.031 to 0.034; intermediate R_s _= 0.018, 95% CI = -0.022 to 0.059; no R_s _= -0.076, 95% CI = -0.317 to 0.169), but significantly positive in low crossover regions (low R_s _= 0.222, 95% CI = 0.165 to 0.284). The fourth chromosome genes show a significantly negative correlation between Fop and d_S _(R_s _= -0.283, 95% CI = -0.502 to -0.022)), whereas for non-fourth chromosome, no crossover genes the relationship is nonsignificantly positive (R_s _= 0.480, 95% CI = -0.013 to 0.776).

Because there has been some suggestion that comparisons of estimates of d_S _from PAML can be misleading when there are large differences in codon usage bias among genes [21], we also examined the relationship between Fop and the nucleotide site-based estimators K_A _and K_S_. Consistent with Bierne and Eyre-Walker [21], the results for K_A _agree very closely with those for d_N _(high R_s _= -0.416, 95% CI = -0.442 to -0.386; intermediate R_s _= -0.456, 95% CI = -0.487 to -0.424; low R_s _= -0.404, 95% CI = -0.456 to -0.350; no R_s _= -0.240, 95% CI = -0.425 to -0.010; fourth R_s _= -0.089, 95% CI = -0.332 to 0.142; non-fourth, no crossover R_s _= -0.123, 95% CI = -0.592 to 0.384). The correlation between Fop and K_S_, however, is quite different from that between Fop and d_S_, being strongly negative in all recombination regions except the no crossover region, where the relationship is not significantly different from zero (high R_s _= -0.377, 95% CI = -0.405 to -0.348; intermediate R_s _= -0.392, 95% CI = -0.425 to -0.359; low R_s _= -0.289, 95% CI = -0.338 to -0.227; no R_s _= 0.194, 95% CI = -0.059 to 0.422; fourth R_s _= 0.096, 95% CI = -0.159 to 0.359; non-fourth, no crossover R_s _= 0.358, 95% -0.118 to 0.743). This is consistent with findings reported by Marais and coworkers [22].

The no crossover region also has a much lower GC content at third position sites (GC3) compared with regions with crossing over (Table 1), as expected from the fact that preferred codons in *D. melanogaster *and its relatives mostly end in G or C [23]. Again, mean GC3 for the non-fourth chromosome genes in the no crossover category is intermediate between the crossing over regions and the genes on the fourth chromosome. If selection for codon usage bias is virtually absent in the no crossover region, then synonymous sites are likely to be evolving close to neutrally. We might therefore expect the GC content at third position sites in the no crossover regions to be closer to equilibrium than in other recombination regions (see Marais and Piganeau [19]).

**Figure 2:**
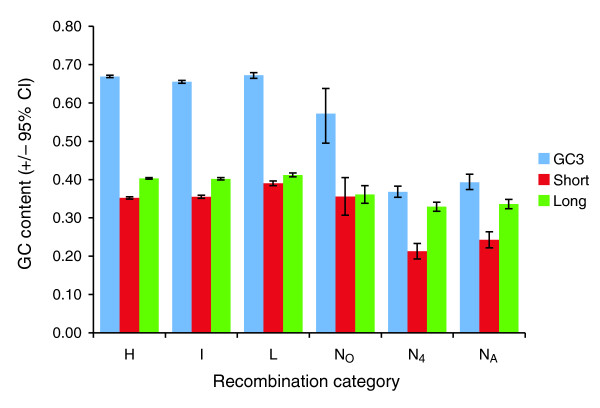
GC content of the third position of codons, short introns, and long introns for each recombination region. GC content at the third position of codons (GC3), short introns (≤80 base pairs [bp]) and long introns (>80 bp) for regions of high, intermediate and low frequency of crossing over and regions of no crossing over. Values reported are means per site for all introns from *D. melanogaster *and *D. yakuba *combined; error bars indicate 95% confidence interval (CI) obtained by bootstrapping by gene/intron.

To examine this, we compared the GC3 values with the GC content in noncoding regions. GC content was calculated for intronic sites in all recombination regions, and the introns were divided into short and long size classes, because these are known to differ dramatically in their rates of evolution [24,25]. Figure 2 and Additional data file 1 show the results of this analysis. Interestingly, the mean GC3 value for the no crossover region (0.39) is similar to the GC content of short introns in other recombination regions (high 0.35, intermediate 0.35, low 0.39). Again, the GC contents of introns in the non-fourth chromosome genes lie between those of the fourth chromosome genes and the rest of the genome. Because short introns represent a class of sites that are likely to be relatively free from selective constraints [25], this suggests that the base composition of third position sites in the no crossover region are indeed closer to neutral equilibrium than those in other recombination regions, as would be expected if the efficacy of selection for codon usage bias were severely limited in this region.

**Figure 3:**
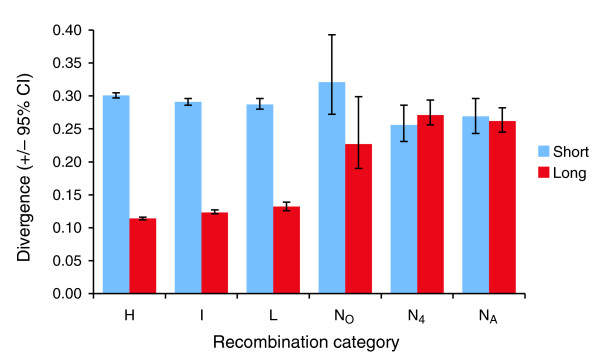
Divergence in short and long introns in each recombination region. Divergence between *D. melanogaster *and *D. yakuba *for short introns (≤80 base pairs [bp]) and long introns (>80 bp) for regions of high, intermediate, and low frequency of crossing over, and regions of no crossing over. Values reported are means per site, corrected for multiple hits [50]. Error bars indicate 95% confidence interval (CI) obtained by bootstrapping by intron.

We also calculated divergence values for short and long introns (omitting sequences that include splice sites) in the different recombination regions, and these show some interesting patterns (Figure 3 and Additional data file 1). Long introns have much lower divergence than short introns, confirming the pattern previously reported between *D. melanogaster *and *D. simulans *introns [24,25]. This pattern is seen in high, intermediate, and low crossing over regions, but not in the no crossover region, where long and short introns exhibit almost identical levels of divergence. This is true when we examine only the fourth chromosome genes; although the non-fourth chromosome genes exhibit lower levels of divergence in long introns than the fourth chromosome genes, there is still a marked increase in intron divergence when comparing regions with crossing over with non-fourth chromosome, no crossover genes.

Previous work also identified a negative relationship between intron length and divergence, and the same pattern is seen here for high, intermediate, and low crossover regions, but not for no crossover regions (Spearman rank correlation [R_s_] with 95% CI [obtained by bootstrapping across introns]: high R_s _= -0.465, 95% CI = -0.481 to -0.450; intermediate R_s _= -0.383, 95% CI = -0.404 to -0.361; low R_s _= -0.322, 95% CI = -0.355 to -0.287; no R_s _= 0.050, 95% CI = -0.095 to 0.188; fourth R_s _= 0.111, 95% CI = -0.066 to 0.262; non-fourth, no crossover R_s _= -0.209, 95% CI = -0.498 to 0.096). The correlation is not significantly different from zero in no crossover regions, and this is true even after using RepeatMasker to mask any microsatellite and/or interspersed repeats (no R_s _= 0.018, 95% CI = -0.132 to 0.158; fourth R_s _= 0.085, 95% CI = -0.074 to 0.251; non-fourth R_s _= -0.245, 95% CI = -0.529 to 0.080; proportion RepeatMasked, including splice sites: no = 0.254; fourth = 0.277; non-fourth, no crossover = 0.152). We further examined this issue by estimating the linear regressions of intron divergence on log intron length for each recombinational environment, because these provide a quantitative estimate of the strength of the relationship; bootstrapping was again used to assess significance. The regression coefficients get closer to zero moving from high to no crossover regions, and are significantly negative in high, intermediate, and low crossover regions, but not in any of the no crossover regions (regression coefficients: high = -0.0503, 95% CI = -0.0518 to -0.0488; intermediate = -0.0423, 95% CI = -0.0448 to -0.0412; low = -0.0356, 95% CI = -0.0384 to -0.0327; no = -0.0008, 95% CI -0.0089 to 0.0068; fourth = 0.0035, 95% CI = -0.0042 to 0.0123; non-fourth, no crossover = -0.0186, 95% CI = -0.0366 to 0.0001). The fact that the regression coefficients are significantly different between high, intermediate, low, and no crossover regions suggests that the efficacy of selection decreases as recombination rate decreases.

Finally, we examined gene length in all recombination regions, because there is evidence suggesting that gene length tends to increase when selective constraints are relaxed [26]. Consistent with this, there was a significant effect of recombination region on gene length (Kruskal-Wallis test: χ^2 ^= 16.71, df = 3, *P *< 10^-3^; Table 1), with genes on the fourth chromosome being longer than those in high, intermediate, and low crossover regions as well as non-fourth chromosome genes in no crossover regions.

## Discussion

One major conclusion from our analysis is that there is a higher rate of nonsynonymous site evolution in the regions of the *Drosophila *genome that apparently lack crossing over, as compared with regions with low to high rates of crossing over (Figure 1). We also found little evidence of differences in d_N _or d_N_/d_S _between low, intermediate, and high crossover regions. This contrasts with the results of Betancourt and Presgraves [12] and Presgraves [13], who found higher nonsynonymous divergence between *D. melanogaster *and *D. simulans *in regions of high recombination when compared with the rest of the genome. The reason for this difference is not entirely clear, but it may reflect the fact that the previous studies were based on relatively few genes. These might have included some genes with unusually high rates of amino acid sequence evolution in the high recombination regions. Consistent with this possibility, Betancourt and Presgraves [12] and Presgraves [13] found a much higher mean ratio of nonsynonymous to synonymous divergence in high recombination regions than in Figure 1. Marais and coworkers [22] also failed to detect any evidence for a positive correlation between the rate of crossing over and nonsynonymous divergence in a comparison of *D. melanogaster *and a set of cDNA sequences from *D. yakuba*; they used similar methods to those of Betancourt and Presgraves [12] and Presgraves [13] to estimate recombination rates, and so the difference in conclusion is unlikely to reflect differences in methods between studies. Rather, as pointed out by Marais and coworkers [22], it is more likely to reflect a bias toward fast-evolving genes in these datasets.

Overall, our results fail to identify faster amino acid sequence evolution in regions of high recombination, but rather they suggest the opposite pattern. They are consistent with less effective selection against weakly deleterious, nonsynonymous mutations when crossing over is effectively absent, as is suggested by studies of the *D. miranda *neo-sex chromosome system [10,11], and as is expected from increased Hill-Robertson effects when crossing over is rare or absent [3,4]. It is, of course, conceivable that the no crossover regions experience a faster rate of adaptive evolution of amino acid mutations, but there is no theoretical basis for expecting this. We also found a significant increase in divergence for long introns (Figure 3) in the no crossover region compared with the rest of the genome; recent studies [24,25] show that longer introns are subject to greater selective constraints than short ones, and so this observation is also consistent with a weakening of selective constraints when recombination rates are very low. Definitive proof of the inference of a relaxation of purifying selection in no crossover regions would require comparisons of within-species polymorphism with between-species divergence, as was done for the *D. miranda *neo-sex chromosome system [11], but suitable data are not yet available.

Another interesting aspect of the results is that the PAML analysis suggests a lower d_S _in the no crossover region of the genome, compared with other regions, although this is not seen in the analysis of K_S _(Figure 1), and we also found a negative relation between Fop and K_S_, but not d_S_, in all but the no crossover regions. This difference between the behavior of the two estimators of synonymous divergence is similar to that found by Bierne and Eyre-Walker [21]. If, as they suggest, estimates of K_S _more accurately reflect divergence at synonymous sites when there are differences in codon usage, our results suggest that selection for codon usage bias acts to reduce divergence at these sites in high, intermediate, and low recombination regions but that this effect is reduced in the absence of crossing over. The K_S _values are thus likely to provide a more reliable indicator of levels of divergence at synonymous sites. For noncoding sites, only K_S _can be used; the results show that divergence in short introns decreases from high to no crossover regions (Figure 3), which is opposite to what is seen for long introns.

There is a slight increase in GC content between high, intermediate, and low crossover regions for short introns (Figure 2), so that the corresponding decrease in short intron divergence might reflect differences in GC to AT mutational biases among these regions, which can cause a negative relationship between divergence and GC content [24]. However, there is a large drop in GC content for short introns in no crossover regions, coupled with a reduction in divergence, which cannot be explained by the mutational bias hypothesis. One possibility is a weakly mutagenic effect of recombination processes, as has been suggested for humans [27]. Ometto and coworkers [28] report a similar pattern for long introns and other noncoding sequences, for divergence between *D. melanogaster *and *D. simulans*. The lack of a similar effect on K_S _for synonymous sites may reflect the fact that the efficacy of selection on codon usage appears to be drastically reduced in the no crossover regions; this would allow a higher rate of synonymous substitutions [21], counter-acting any effect of reduced recombination on mutation rates.

There has been some controversy over the negative correlation between GC content/codon usage and rate of crossing over in the *D melanogaster *genome, which has been reported in previous studies. Marais and coworkers [19,29,30] argued that this correlation mainly reflects the effect of differences in mutational bias and/or the rate of biased gene conversion (BGC) in favor of GC versus AT, which should affect putatively neutral noncoding sequences, whereas Kliman and Hey [20] and Hey and Kliman [31] argued for an effect of reduced recombination on the efficacy of selection. These analyses used longer introns to estimate the effects of mutational bias and BGC, on the grounds that these are less likely to be affected by selective constraints on splice sites and hence evolve neutrally. As we have seen, this assumption is probably incorrect. Our results for short introns outside the no crossover regions show, if anything, the opposite pattern to that expected based on the BGC hypothesis, because for short introns there is a slight decrease in divergence and increase in GC content between high and low crossover regions. Long introns exhibit almost no differences in GC content moving from high to low crossover regions, but they show a slight increase in divergence. Their GC content drops substantially in the no crossover regions. This behavior of the GC content of long introns is similar to that reported by Kliman and Hey [20]. Long introns, but not short ones, exhibit a large increase in divergence in the no crossover regions (Figure 3), which is consistent with a relaxation of selective constraints. GC content at third coding positions is still higher than for introns, even in the no crossover regions (Figure 2), suggesting that there is still some selection in favor of preferred codons in these regions.

Overall, these patterns suggest that selective constraints on weakly deleterious amino acid mutations, mutations to nonpreferred codons, and weakly deleterious mutations in long introns are reduced in genomic regions where crossing over is virtually absent, but they are little affected by rates of crossing over in other regions. One caveat concerning our conclusions is that the recombinational landscape may well differ between *D. melanogaster *and *D. yakuba*, for which there is some evidence [32]. As described in Materials and methods (below), we have attempted to eliminate genes that differ between the species with respect to their location in telomeric and centromeric regions, where crossing over is absent or greatly reduced [22]. However, we cannot exclude smaller differences between species in recombination patterns. Such differences may be why there is little or no effect of crossing over rate in regions of low to high recombination, despite the fact that these are known to show clear patterns with respect to neutral diversity in *D. melanogaster *[13,28]. *D. melanogaster *might have only relatively recently evolved low recombination over more extensive regions than in its common ancestor with *D. yakuba*. Because codon usage, GC content, and divergence must change over longer time scales than neutral diversity within species, this could account for the discrepancy between the pattern for diversity and the other statistics.

The other possibility is that there is a strongly nonlinear effect of recombination on Hill-Robertson effects. This does not seem likely for either selective sweep or background selection processes [33,34], but it does apply to Muller's ratchet [35] and Hill-Robertson interference among groups of weakly selected sites [36]. However, it is unclear whether these effects would be strong enough to explain the patterns that we observe.

There is a general tendency for the effects that we detect to involve primarily differences between chromosome four and the rest of the genome, such that effects on genes with no crossing over that are located on other chromosomes appear to be weak or absent (Table 1 and Figures 1, 2, 3). This may well reflect the fact that the fourth chromosome is a block of more than 80 genes that fail to crossover with each other, whereas there are much smaller numbers of genes in the no crossover regions of the other chromosomes. There is thus much less opportunity for enhanced Hill-Robertson effects on the latter. It is noteworthy that this difference between the fourth chromosome and other no crossover regions is most marked for the rate of nonsynonymous substitution, for which selective constraints are likely to be stronger and hence more resistant to a moderate reduction in effective population size [34]. Alternatively, this pattern may reflect the fact that, although chromosome four has a stable history of no crossing over, it is unclear whether this is true of the no crossover regions on other chromosomes (see above).

There is one other apparent anomaly in the results, which at first sight is difficult to explain. This is the extreme reduction in GC content of short introns in the no crossover regions (Figure 2), to a mean level that is lower than that of long introns, despite the evidence for a reduced effectiveness of selection in these regions. This may reflect a drastic reduction in the intensity or efficacy of BGC in favor of GC in this region, because this would be the only deterministic force affecting neutral sequences, whereas long introns and synonymous sites are subject to both selection and BGC. This is seemingly inconsistent with the similarity in divergence for long and short introns in the no crossover regions. However, with very weak selection there can be interactions between mutational bias and the product of effective population size and selection coefficient, causing substitution rates to be almost flat or even increase with N_e_s in regions where N_e_s is very small [34,37-39]. Thus, it is theoretically possible for long introns to be under effectively stronger selection than short introns, but to show the same or even higher levels of divergence in no crossover regions.

## Conclusion

We have examined the effect of recombinational environment, in terms of the frequency of crossing over, on the rates of nonsynonymous and synonymous evolution, codon usage bias, and evolution of noncoding DNA. Although we find only very small differences between regions of high, intermediate, and low crossing over frequency, the absence of crossing over appears to have a profound effect on patterns of molecular evolution. The no crossover regions exhibit elevated levels of nonsynonymous evolution, a virtual absence of codon usage bias, and similar levels of divergence for short and long intron size classes. These patterns are all consistent with a dramatic reduction in the efficacy of selection in the absence of crossing over, as a result of greatly enhanced effects of Hill-Robertson interference.

## Materials and methods

### Fourth chromosome data

FlyBase [40] was used to download a list of all *D. melanogaster *genes with cytological map locations in bands 101 and 102. The genome annotation for each of these genes was examined, and any genes without expressed sequence tag or cDNA hits, or without any genome annotation, were eliminated. For the remaining genes, decorated fasta files containing coding regions were downloaded from FlyMine [41]. Where genes are not alternatively spliced, the entire coding region was used in the analysis. For genes that are alternatively spliced, only constitutively spliced exons were used. Exons were also eliminated if they overlapped with coding sequence on the opposite strand. Homologous sequences from *D. yakuba *were found using BLAST searches on the DroSpeGe website [42], and individual exons aligned by eye using Sequencher (Gene Codes, Ann Arbor, MI, USA). Exons were then concatenated and a fasta file containing the entire coding region for both species was exported for each locus. These fasta files were then aligned and analyzed as described below for the non-fourth chromosome data.

### Non-fourth chromosome data

In order to generate alignments of constitutively spliced exons from coding sequences between *D. melanogaster *and *D. yakuba*, we used a modified version of the methods described by Halligan and Keightley [25]. This involved obtaining a list of all currently annotated *D. melanogaster *genes from NCBI's Entrez Gene (using release 4.1 of the *D. melanogaster *genome), giving a total of 14,183 annotations. From this list, RNA genes and poorly annotated genes were excluded by examining the Flybase synopsis report for each gene, and excluding genes that were based on BLASTX data or gene prediction data only. Genbank format files were then downloaded for the remaining genes (including all annotated spliceforms), to give a dataset of 11,267 Genbank files. We extracted all annotated exons for a randomly chosen spliceform from each gene and used a reciprocal best-hits BLAST approach to identify and extract orthologous exons from the November 2005 freeze of the *D. yakuba *genome sequence (Genome Sequencing Center, Washington University School of Medicine, St Louis, MI, USA. Short exons (<40 bp) were joined, where possible, to an adjacent section of noncoding DNA (either intronic or intergenic) prior to BLASTing, to increase the chance of a reciprocal best-hit. We used the locations of the orthologous exons in the draft *D. yakuba *genome sequence to retrieve the orthologous intron sequences. Introns were only retrieved if two adjacent *D. melanogaster *exons were identified on the same strand and same contig in the *D. yakuba *genome.

Coding sequences (formed by concatenating the retrieved exons from the chosen spliceform) from both species were aligned using the amino acid alignment obtained from CLUSTALW [43]. Genes were removed from the data set if the coding sequence was invalid in either species. A coding sequence was considered to be valid if it started with a start codon, ended with a stop codon, was a multiple of 3 bp in length, and contained no internal stop codons. We removed exons that were not constitutively spliced (not present in every annotated spliceform) from the coding sequences and ensured that the remaining exons were in frame and a multiple of 3 bp in length. Introns were initially aligned using MAVID [44] and were subsequently realigned at a finer scale using MCALIGN2 [45] by splitting the MAVID alignments into sections of approximately 500 bp at regions of high homology (>8 bp runs of ungapped matches). Introns were removed from the dataset if the sequence in either species did not start and end with a 2 bp consensus sequence ('AT', 'GT', or 'GC' at the 5' end and 'AG' at the 3' end). All alignments (both intronic and coding) with fewer than 10 valid bases (A, T, G, or C) or fewer than 20 valid/invalid bases (A, T, G, C, or N) in either species were discarded. Any clearly nonhomologous sections were masked from all alignments (defined as regions where divergence was above 0.25 within a 40 to 60 bp sliding window).

### Estimating measures of divergence and codon usage bias

Maximum-likelihood estimates of d_N _and d_S _for each gene were obtained using Codeml in the PAML package [46], using runmode = -2. Because estimates of d_N_/d_S _are likely to be unreliable for very short genes, alignments less than 150 bp (50 codons) in length were removed. We also used Gestimator [47], which implements the method of Comeron [48], to calculate values of K_A _and K_S_. Estimates of ENC and Fop were calculated using codonw [49]. GC content was estimated both for the entire coding sequence and for the third positions of codons only (GC3). We also estimated GC content and divergence (corrected for multiple hits [50]) in introns, following removal of 8 bp/30 bp at the beginning/end of the introns to exclude any sites that may be subject to selective constraints [25].

### Recombination regions

The entire dataset was sorted according to cytologic map location, and was divided into groups with high, intermediate, and low frequencies of crossing over, and a group with no crossing over, based on the regions described by Charlesworth [51] (Additional data file 2). In addition to this, data from a number of cytologic bands were eliminated from the analysis, as described by Marais and coworkers [22]. This removes genes in telomeric and centromeric polytene bands that have shifted in position between *D. melanogaster *and *D. yakuba*, and hence will have experienced a major change in recombinational environment.

## Additional data files

The following additional data files are available with the online version of this article. Additional data file 1 contains information on mean values of GC content and divergence for short and long intron classes in the different recombination regions. Additional data file 2 contains information on the division of data into recombination classes based on cytologic location.

## Supplementary Material

Additional data file 1 Information on mean values of GC content and divergence for short and long intron classes in the different recombination regions Click here for file

Additional data file 2 Information on the division of data into recombination classes based on cytologic location Click here for file

## Figures and Tables

**Table 1:** Measures of codon usage bias, GC content, and gene length in the different recombination regions

	ENC	Fop	GC total	GC3	Length
High	47.41 (47.23-47.62)	0.545 (0.542-0.547)	0.548 (0.546-0.549)	0.669 (0.666-0.672)	1517 (1477-1562)
Intermediate	48.57 (48.33-48.83)	0.532 (0.528-0.535)	0.541 (0.538-0.543)	0.655 (0.651-0.659)	1476 (1424-1520)
Low	47.86 (47.44-48.25)	0.548 (0.543-0.554)	0.549 (0.546-0.552)	0.672 (0.664-0.679)	1489 (1422-1556)
No (N_O_)	52.24 (49.69-54.42)	0.424 (0.378-0.467)	0.511 (0.466-0.547)	0.572 (0.495-0.638)	1238 (915-1590)
No (N_4_)	54.14 (53.43-54.82)	0.263 (0.251-0.276)	0.422 (0.411-0.432)	0.368 (0.354-0.383)	2692 (2053-3532)
No (N_A_)	53.70 (52.89-54.50)	0.300 (0.280-0.321)	0.432 (0.421-0.446)	0.393 (0.374-0.414)	2358 (1860-3016)

Values reported for ENC, Fop, GC total, and GC3 are means for all genes from *D. melanogaster *and *D. yakuba *combined (95% confidence interval). Values for gene length are the mean number of base pairs in *D. melanogaster *for all constitutively spliced exons concatenated, for each gene (95% confidence interval). The no crossing over region is divided as follows: N_O_, non-fourth chromosome genes; N_4_, fourth chromosome genes; and N_A_, all no crossing over region genes. ENC, effective number of codons; Fop, frequency of optimal codons; GC3, GC content of the third position of codons.
