# Supplementary material for: Reduced efficacy of selection in regions of the Drosophila genome that lack crossing over
Source: Genome Biol. 2007 Feb 6;8(2):R18. doi: 10.1186/gb-2007-8-2-r18 (PMC1852418; doi:10.1186/gb-2007-8-2-r18)
Supplement: Additional data file 1 — Information on mean values of GC content and divergence for short and long intron classes in the different recombination regions [file gb-2007-8-2-r18-S1.pdf]

Additional data file 1: GC content and divergence for short (≤80 bp) and long (>80 bp) intron classes in the different recombination regions.

Rec. region	Intron size class	GC content	Divergence
High	Short	0.352 (0.349/0.355)	0.301 (0.297/0.305)
	Long	0.403 (0.400/0.405)	0.114 (0.112/0.116)
Intermediate	Short	0.355 (0.350/0.359)	0.291 (0.286/0.296)
	Long	0.402 (0.399/0.405)	0.123 (0.121/0.127)
Low	Short	0.390 (0.385/0.396)	0.287 (0.280/0.296)
	Long	0.412 (0.408/0.417)	0.132 (0.126/0.139)
N_O	Short	0.356 (0.304/.0405)	0.321 (0.272/0.393)
	Long	0.361 (0.320/0.384)	0.227 (0.190/0.299)
N_4	Short	0.213 (0.195/0.233)	0.256 (0.231/0.286)
	Long	0.329 (0.315/0.341)	0.271 (0.256/0.294)
N_A	Short	0.243 (0.220/0.264)	0.269 (0.243/0.296)
	Long	0.336 (0.321/0.348)	0.262 (0.245/0.282)

Values reported are means per site, corrected for multiple hits [50], with 95% confidence intervals obtained by bootstrapping by intron. The no crossing over region is divided as follows: N_O = non-fourth chromosome genes, N_4 = fourth chromosome genes, N_A = all no crossing over region genes.
