# Supplementary material for: Reduced efficacy of selection in regions of the Drosophila genome that lack crossing over
Source: Genome Biol. 2007 Feb 6;8(2):R18. doi: 10.1186/gb-2007-8-2-r18 (PMC1852418; doi:10.1186/gb-2007-8-2-r18)
Supplement: Additional data file 2 — Information on the division of data into recombination classes based on cytologic location [file gb-2007-8-2-r18-S2.pdf]

Additional data file 2: Division of data into recombination classes, based on cytological location [see 51]

Chromosome	Cytological location	Sequence coordinates	Recombination class	Gene boundaries [a]
X	Up to 1B4	1 – 342892	No	None
	1B5 to 3C2	342892 – 2764070	Low	CG13366 / CG14419
	3C3 to 15F1-3	2764070 – 17047156	High	CG14423 / CG12995
	15F4 to 19D3	17047156 – 20415661	Intermediate	CG5162 / CG18492
	19E1 to 20C1	20415661 – 21887054	Low	CG1829 / CG17599
	20C2 to 20F4	21887054 – 22714666	No	CG12446 / CG9559
2L	Up to 22A1	-204334 – 1428804	Low	CG11912 / CG31659
	22A2 to 31A1	1428804 – 10056945	High	CG33128 / CG13127
	31A2 to 38A1	10056945 – 19717726	Intermediate	CG4799 / CG10443
	38A2 to 40C1	19717726 – 21934144	Low	CG10628 / CG11634
	40C2 to 40F7	21934144 – 22557853	No	CG10834 / CG12775
2R	Up to 41E1	-31698 – 743919	No	None

	41E2 to 42F3	743919 – 2692486	Low	CG2682 / CG11060
	43A1 to 50F9	2692486 – 9904354	Intermediate	CG30385 / CG8422
	51A1 to 59F8	9904354 – 19294758	High	CG10104 / CG5532
	60A1 to 60F5	19294758 – 20941487	Low	CG13561 / CG30429
3L	Up to 62A12	-315927 – 1694342	Low	CG6821 / CG12011
	62B1 to 71A1	1694342 – 14853328	High	CG18171 / CG17839
	71A2 to 77E1	14853328 – 20578766	Intermediate	CG13466 / CG4786
	77E2 to 80A1	20578766 – 22695409	Low	CG4717 / CG32451
	80A2 to 80F9	22695409 – 23790248	No	CG14448 / CG32230
3R	Up to 81F2	-224037 – -132221	No	None
	81F3 to 84B1	-132221 – 2811817	Low	CG18090 / CG2047
	84B2 to 89F4	2811817 – 13082065	Intermediate	CG1982 / CG4135
	90A1 to 99F1	13082065 – 26191519	High	CG31419 / CG18404
	99F2 to 100F5	26191519 – 28686604	Low	CG1469 / CG1483

4	101F1 to 102F8	9362 – 1984823	No	CG17245 / CG33653

[a] indicates the distal and proximal genes analysed in each of the recombination categories.
